# A CCO–PPO Framework for Autonomous UAV Trajectory Tracking in Complex and Disturbed Environments

**DOI:** 10.3390/s26092735

**Published:** 2026-04-28

**Authors:** Xize Guo, Chao Fan, Boxuan Shao, Qi Deng, Jiahao Chen, Tao Zhang, Wentao Zhang

**Affiliations:** 1 National Elite Institute of Engineering, Northwestern Polytechnical University, Xi’an 710072, China; 2 School of Software, Northwestern Polytechnical University, Xi’an 710072, China; 3 Capital University of Economics and Business, Beijing 100070, China; 4 Chongqing Chang’an Industrial (Group) Co., Ltd. Shenzhen Branch, Shenzhen 518000, China; 5 School of Information Engineering, Capital Normal University, Beijing 100048, China

**Keywords:** unmanned aerial vehicle, trajectory tracking, proximal policy optimization, hyperparameter optimization, cuckoo catfish optimizer, metaheuristic algorithm, reinforcement learning

## Abstract

Accurate trajectory tracking is fundamental to the autonomous operation of unmanned aerial vehicles (UAVs) in complex tasks. While proximal policy optimization (PPO) has shown strong potential in UAV control, its performance is highly sensitive to hyperparameter configuration, and manual tuning is time-consuming due to complex interparameter coupling. This paper proposes CCO–PPO, a framework integrating the cuckoo catfish optimizer (CCO) with PPO for automatic hyperparameter optimization in UAV trajectory tracking. The problem is formulated as a Markov decision process with a 20-dimensional state space, and the CCO performs offline search over a four-dimensional hyperparameter space. Evaluated across seven test environments covering diverse trajectory geometries, wind disturbances, sensor noise, and large-scale scenarios, CCO–PPO achieves the lowest tracking error in all cases. Performance gains over baseline PPO increase monotonically with task complexity, reaching 18.8% under combined wind disturbance and sensor noise, with statistically significant advantages in 85.7% of pairwise comparisons against baseline PPO, SAC, and TD3. Ablation studies confirm that joint optimization of all four hyperparameters is essential under high-disturbance conditions, and comparisons with Bayesian optimization validate the CCO’s superior cross-seed stability. These results demonstrate that metaheuristic hyperparameter optimization substantially enhances policy robustness in high-disturbance UAV trajectory tracking scenarios.

## 1. Introduction

Unmanned aerial vehicles (UAVs), owing to their flexibility and autonomy, have demonstrated broad application prospects in search and rescue, aerial surveying and mapping, precision agriculture, and autonomous delivery, where high-precision trajectory tracking is fundamental to mission success. Reinforcement learning-based control methods, particularly the proximal policy optimization (PPO) algorithm, have emerged as a promising direction in UAV trajectory tracking due to their model-free end-to-end learning advantages. Nevertheless, PPO performance is highly sensitive to the learning rate, clipping parameter ε, discount factor γ, and GAE parameter λ, whose complex coupling makes manual tuning time-consuming and prone to poor generalization under complex disturbances. Metaheuristic optimization algorithms, through population-based global search mechanisms, offer an effective pathway for automatic hyperparameter tuning.

To this end, this paper proposes the CCO–PPO framework, which integrates the chaotic cuckoo catfish optimization algorithm (CCO)—a swarm intelligence algorithm proposed in 2025 that employs a three-phase search mechanism and death-and-parasitism strategy—with PPO for offline automatic search over a four-dimensional hyperparameter space.

The framework formulates trajectory tracking as an MDP and employs a multi-component reward function covering position, velocity, and attitude. Across seven test environments encompassing diverse geometric trajectories, wind disturbances, sensor noise, and large-scale generalization scenarios, CCO–PPO achieves the lowest tracking error under all the conditions, attaining a performance improvement of up to 18.8% over the baseline PPO under the extreme conditions of combined wind disturbance and sensor noise while also demonstrating favorable scale generalization capability.

## 2. Related Work

### 2.1. Reinforcement Learning-Based UAV Trajectory Tracking

UAV trajectory tracking control methods can be broadly categorized into two classes: model-based traditional approaches and learning-based modern approaches. Traditional methods such as PID control, Model Predictive Control (MPC), and Linear Quadratic Regulator (LQR) rely on precise dynamic models and exhibit limited robustness under nonlinear dynamics and external disturbances [1,2]. In recent years, deep reinforcement learning (DRL) has achieved remarkable progress in UAV control owing to its model-free end-to-end learning advantages [3]. PPO, as an on-policy actor–critic algorithm with clipped policy updates, has been widely applied to attitude control and trajectory tracking of quadrotor UAVs [4]. SAC achieves exploration–exploitation balance via automatic entropy tuning [5], while TD3 mitigates overestimation through delayed updates and target smoothing [6]—both demonstrating strong performance in continuous control tasks. Nevertheless, all these algorithms remain sensitive to hyperparameter configurations, with manual tuning becoming increasingly impractical in complex UAV scenarios [7].

In the domain of UAV three-dimensional path planning, metaheuristic algorithms have demonstrated broad application potential. Hu et al. proposed the MAHACO, which incorporates differential evolution mutation strategies into an ant colony optimization framework to achieve efficient search for smooth 3D paths in multi-UAV scenarios [8]; Santos et al. improved path quality in three-dimensional voxel maps by extending the neighborhood structure of A*, Theta*, and JPS algorithms beyond the standard 26-adjacency, achieving a more favorable trade-off between path quality and computational overhead [9]; Xu et al. proposed the DBO–AWOA, integrating chaotic initialization and dung beetle optimizer-inspired reproductive mechanisms into the whale optimization algorithm, generating smoother and more energy-efficient UAV paths in three-dimensional mountainous environments [10]. However, these works optimize waypoint sequences for offline path planning, which is fundamentally distinct from the online trajectory tracking problem addressed here, where metaheuristic algorithms are applied to RL hyperparameter optimization.

### 2.2. Hyperparameter Optimization for Reinforcement Learning

Hyperparameter optimization is one of the key factors influencing the performance of reinforcement learning algorithms. The existing methods primarily include grid search, random search [11], Bayesian optimization (BO), and Population-Based Training (PBT). Grid search and random search are straightforward to implement but incur prohibitive computational costs in high-dimensional search spaces [11]; BO achieves relatively high sample efficiency via surrogate models, yet its accuracy is limited for high-dimensional non-stationary problems [12]; PBT dynamically adjusts hyperparameters during training but incurs substantial overhead from maintaining a large population [13]. Metaheuristic algorithms such as particle swarm optimization (PSO) [14], differential evolution (DE) [15], and the dung beetle optimizer (DBO) [16], which search complex non-convex spaces via nature-inspired collective behaviors, have been progressively introduced into RL hyperparameter tuning, demonstrating favorable global search capability and scalability. However, the existing studies predominantly focus on standard benchmark tasks, and systematic hyperparameter optimization research for practical tasks with high-dimensional state spaces, continuous action control, and strong disturbance characteristics—such as UAV trajectory tracking—remains scarce [7,16].

## 3. Method

### 3.1. Problem Formulation and Modeling

#### 3.1.1. Formalization of the UAV Trajectory Tracking Problem

**Figure 1:**
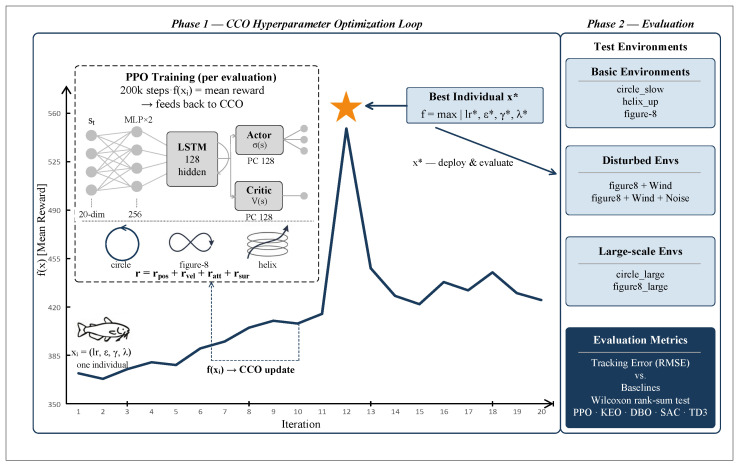
Overview of the CCO–PPO framework for UAV trajectory tracking hyperparameter optimization.

The UAV trajectory tracking problem is formulated as a Markov decision process (MDP) [17,18], defined as a quintuple M=(S,A,P,R,γ), where S denotes the state space, A denotes the action space, P:S×A×S→[0,1] is the state transition probability function, R:S×A→R is the reward function, and γ∈[0,1) is the discount factor. The overall framework is illustrated in Figure 1.

**State Space Definition.** To jointly address position accuracy, velocity matching, and attitude stability, a 20-dimensional observation space $S\in R^{20}$ is designed, comprising

$$s_{t}=\left[\Delta p_{t},\;p_{t},\;v_{t},\;\Delta \phi_{t},\;\omega_{t},\;p_{t}^{\mathrm{target}},\;v_{t}^{\mathrm{target}}\right]$$

where $\Delta p_{t}={\left[dx,\;dy,\;dz\right]}^{⊤}\in R^{3}$: position error between UAV’s current and target positions; $p_{t}={\left[x,\;y,\;z\right]}^{⊤}\in R^{3}$: absolute position of UAV in inertial frame; $v_{t}={\left[v_{x},\;v_{y},\;v_{z}\right]}^{⊤}\in R^{3}$: linear velocity of UAV; $\Delta \phi_{t}={\left[\Delta \phi ,\;\Delta \theta \right]}^{⊤}\in R^{2}$: roll/pitch attitude deviations; $\omega_{t}={\left[\omega_{x},\;\omega_{y},\;\omega_{z}\right]}^{⊤}\in R^{3}$: angular velocity of UAV; $p_{t}^{\mathrm{target}}={\left[x_{t},\;y_{t},\;z_{t}\right]}^{⊤}\in R^{3}$: target position on reference trajectory (current time step); $v_{t}^{\mathrm{target}}={\left[v_{tx},\;v_{ty},\;v_{tz}\right]}^{⊤}\in R^{3}$: target velocity of reference trajectory.

This state representation integrates error and absolute information, enabling the policy to perceive tracking deviations while retaining global position and target motion trends.

**Action Space Definition.** This paper adopts a continuous action space $A\in {\left[-1,1\right]}^{4}$, with the action vector defined as

$$a_{t}={\left[u_{\mathrm{thrust}},\;u_{\mathrm{roll}},\;u_{\mathrm{pitch}},\;u_{\mathrm{yaw}\_\mathrm{rate}}\right]}^{⊤}$$

where $u_{\mathrm{thrust}}$ is the normalized thrust command, $u_{\mathrm{roll}}$ and $u_{\mathrm{pitch}}$ are the roll and pitch angle control commands, and $u_{\mathrm{yaw}\_\mathrm{rate}}$ is the yaw rate command. All action components are mapped to actual control inputs via a low-level controller.

**Reward Function Design.** To achieve high-precision trajectory tracking, the reward function must jointly account for position error, velocity matching, and attitude stability. The reward function designed in this paper takes the form

$$r_{t}=-\lambda_{p}∥\Delta p_{t}∥-\lambda_{v}∥v_{t}-v_{t}^{\mathrm{target}}∥-\lambda_{\phi }∥\Delta \phi_{t}∥-\lambda_{\omega }∥\omega_{t}∥$$

where $\lambda_{p},\lambda_{v},\lambda_{\phi },\lambda_{\omega }$ are weighting coefficients and ∥·∥ denotes the Euclidean norm. The four terms penalize position error, velocity deviation, attitude deviation, and excessive angular velocity, respectively.

The optimization objective of the trajectory tracking problem is to find an optimal policy π* that maximizes the expected cumulative reward:

$$\pi *=\arg\max_{\pi }E_{\tau ∼\pi }\left[\sum_{t=0}^{T}\gamma^{t}r_{t}\right]$$

where $\tau =(s_{0},a_{0},s_{1},a_{1},\ldots )$ denotes the trajectory generated by policy π.

#### 3.1.2. PPO-Based Reinforcement Learning Solution Framework

PPO is employed to solve the formulated MDP, adopting an actor–critic architecture in which the actor outputs action distribution $\pi_{\theta }\left(a|s\right)$ and the critic estimates state value $V_{\phi }\left(s\right)$ [4]. Both networks use an MLP–LSTM structure to capture temporal dependencies in trajectory tracking.

**Policy Update.** At each control time step *t*, PPO updates the policy parameters θ by maximizing the following clipped surrogate objective:

$$L^{CLIP}\left(\theta \right)=E_{t}\left[\min\left(r_{t}\left(\theta \right){\hat{A}}_{t}^{uav},\;\mathrm{clip}\left(r_{t}\left(\theta \right),\;1-\epsilon ,\;1+\epsilon \right){\hat{A}}_{t}^{uav}\right)\right]$$

where $r_{t}\left(\theta \right)=\frac{\pi_{\theta }\left(a_{t}|s_{t}\right)}{\pi_{\theta_{\mathrm{old}}}\left(a_{t}|s_{t}\right)}$, and ${\hat{A}}_{t}^{uav}$ is the advantage estimate reflecting the contribution of action $a_{t}$ relative to the baseline policy.

**Advantage Function Estimation.** Based on GAE [19], the advantage is computed using the tracking reward $r_{t}$:

$${\hat{A}}_{t}^{uav}=\sum_{l=0}^{\infty }{\left(\gamma \lambda \right)}^{l}\left(r_{t+l}+\gamma V_{\phi }\left(s_{t+l+1}\right)-V_{\phi }\left(s_{t+l}\right)\right)$$

where $r_{t+l}=-\lambda_{p}∥\Delta p_{t+l}∥-\lambda_{v}∥v_{t+l}-v_{t+l}^{\mathrm{target}}∥-\lambda_{\phi }∥\Delta \phi_{t+l}∥-\lambda_{\omega }∥\omega_{t+l}∥$, and γ and λ trade off bias and variance in future tracking error estimation.

**Value Function Update.** The critic network updates parameters ϕ by minimizing the following loss:

$$L^{VF}\left(\phi \right)=E_{t}\left[{\left(V_{\phi }\left(s_{t}\right)-\left({\hat{A}}_{t}^{uav}+V_{\phi }\left(s_{t}\right)\right)\right)}^{2}\right]$$

where $V_{\phi }\left(s_{t}\right)$ estimates the expected cumulative discounted reward from state $s_{t}$ under policy $\pi_{\theta }$.

**Hyperparameter Sensitivity.** Given the complex coupling among these hyperparameters and the limitations of manual tuning, a metaheuristic algorithm is introduced to perform automatic search over the four hyperparameters.

#### 3.1.3. Evaluation Metrics

This paper evaluates algorithm performance along three dimensions: task completion quality, training stability, and statistical significance.

**Mean Reward.** The expected total reward over an entire episode:

$$\bar{R}=\frac{1}{n}\sum_{i=1}^{n}R_{i}$$

**Mean Tracking Error.** Quantifies the spatial deviation between the UAV’s actual trajectory and the reference trajectory:

$$\bar{e}=\frac{1}{T}\sum_{t=1}^{T}∥p_{t}-p_{t}^{\mathrm{target}}∥$$

where *T* is the total number of steps per episode. This metric serves as the primary performance measure in the comprehensive validation experiment (Section 4.4).

**Standard Deviation.** Evaluates algorithm stability across random seeds:

$$\sigma =\sqrt{\frac{1}{n-1}\sum_{i=1}^{n}{\left(x_{i}-\bar{x}\right)}^{2}}$$

Results are reported as mean ± standard deviation, with smaller σ indicating better stability.

**Significance Testing.** The Wilcoxon rank-sum test (α=0.05) is employed to assess the statistical significance of performance differences between each comparison algorithm and CCO–PPO and is well suited to the small-sample setting (n=5) of this study.

#### 3.1.4. Comparison Algorithms and Parameter Configurations

To comprehensively evaluate the performance of CCO–PPO, this paper establishes the following comparison algorithms and parameter configurations.

**Baseline PPO.** The standard hyperparameters from the original PPO paper [4] are adopted as the baseline: $lr=3\times 10^{-4}$, ϵ=0.2, γ=0.99, and λ=0.95.

**Metaheuristic-Optimized PPO Variants.** The hyperparameter optimization experiment (Section 4.2) compares four configurations: CCO–PPO, DBO–PPO, KEO–PPO, and PSO–PPO. All four algorithms optimize the same four hyperparameters with shared settings: population size N=6, maximum number of iterations MaxIt=20, and stagnation threshold of 10 generations. Algorithm-specific parameters are as follows: the CCO uses spiral parameters α=1.34 and $\beta_{s}=0.3$, Lévy flight parameter β=1.5, and a stagnation switching interval of 15 generations; the KEO uses a sub-swarm ratio of 0.05 and an energy threshold of 0.5; PSO uses an inertia weight linearly decaying from 0.9 to 0.4, cognitive coefficient $c_{1}=2.0$, and social coefficient $c_{2}=2.0$.

**Off-Policy Comparison Algorithms.** The comprehensive validation experiment (Section 4.4) introduces SAC and TD3 to evaluate performance differences between on-policy and off-policy algorithms on trajectory tracking tasks. Both SAC and TD3 share: lr $3\times 10^{-4}$, batch size 256, replay buffer capacity 100,000, γ=0.99, soft update coefficient τ=0.005, learning start 10,000 steps, and two FC layers of 256 units. TD3-specific parameters include: policy delayed update frequency of 2, target policy noise of 0.2, noise clipping range of 0.5, and exploration noise of 0.1. SAC employs an automatic entropy adjustment mechanism.

**Shared PPO Parameter Configuration.** All PPO variants share the following settings to ensure fair comparison. Training hyperparameters: PPO update epochs ppo_epoch = 10, number of mini-batches 4, data chunk length 10, value loss coefficient 0.1, entropy coefficient 0.05, and gradient clipping threshold 0.5. Network architecture: two 256-dimensional MLP layers followed by a single 128-dimensional LSTM layer as the shared feature extractor, with a 128-dimensional fully connected layer appended to each of the actor and critic heads, ReLU activation functions, and feature normalization with clipped value loss. Training employs 8 parallel environments for synchronous experience collection, with a buffer size of 200 steps per environment.

**Evaluation Configuration.** All algorithms are evaluated every 5000 training steps, with 20 episodes per evaluation run, using a deterministic policy. In the hyperparameter optimization experiment (Section 4.2), each configuration is independently run 5 times (15 times for the baseline); in the comprehensive validation experiment (Section 4.4), CCO–PPO selects the 5 optimal hyperparameter configurations identified in Section 4.2, each run 5 times with different random seeds for a total of 25 runs, while all other algorithms are each run 5 times.

### 3.2. CCO-Based PPO Hyperparameter Optimization Method

#### 3.2.1. Cuckoo Catfish Optimizer

**Figure 2:**
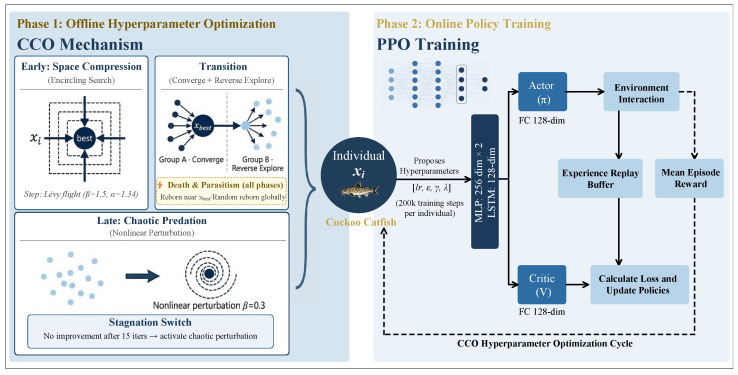
Detailed illustration of the CCO–PPO framework: Phase 1—CCO hyperparameter optimization mechanism; Phase 2—PPO policy training pipeline.

The cuckoo catfish optimizer (CCO) is a swarm intelligence metaheuristic algorithm proposed by Wang et al. in 2025, published in Artificial Intelligence Review (SCI Q1) [20], which balances global exploration and local exploitation by modeling the parasitic reproduction and predatory behaviors of the cuckoo catfish, as illustrated in Figure 2.

The CCO divides the optimization process into three phases. In the early phase, a space-compression and encircling search strategy drives broad population coverage. In the middle phase, the population splits into two groups—one converging toward the global optimum and the other performing reverse exploration. In the late phase, a chaotic predation strategy applies nonlinear perturbations near the current best solution for fine-grained exploitation [20]. A death-and-parasitism mechanism runs throughout all phases, replenishing diversity through parasitic rebirth near the optimum or global random rebirth to escape local optima.

Compared with other metaheuristics evaluated in this study, the CCO offers distinct advantages for this task. PSO relies on a single velocity-update rule without phase transitions, limiting adaptability when the fitness landscape is non-convex and stochastic—as is inherently the case when PPO outcomes vary across random seeds. The DBO provides adequate local exploitation but lacks a dedicated global exploration mechanism, leading to conservative behavior in high-dimensional spaces. The KEO’s performance is sensitive to initialization, risking inconsistent results across seeds. By contrast, the CCO’s death-and-parasitism mechanism continuously replenishes population diversity throughout all phases, making it particularly robust to the stochastic noise in fitness evaluation inherent to PPO’s on-policy training dynamics.

The CCO treats each hyperparameter configuration [lr,ϵ,γ,λ] as an individual in a four-dimensional search space. The fitness function is defined as the mean cumulative reward after training a PPO algorithm for 200,000 steps in the circle_slow environment, with N=6 and MaxIt=20, yielding 120 configurations evaluated in total.

#### 3.2.2. CCO–PPO Hyperparameter Optimization Framework

CCO–PPO integrates the CCO with PPO for automatic hyperparameter optimization via an offline strategy, completing the search prior to full policy training. The CCO operates in a four-dimensional continuous search space, where each individual’s position vector corresponds to a set of PPO hyperparameters [lr,ϵ,γ,λ]; the learning rate is mapped on a logarithmic scale, while ϵ, γ, and λ are mapped linearly, ensuring consistency between the search space and the actual hyperparameter ranges.

The complete execution pipeline consists of three phases. Phase 1—hyperparameter optimization: the CCO iteratively searches within the training environment circle_slow, where the hyperparameter configuration corresponding to each individual requires training a PPO model for 200,000 steps, evaluated every 5000 steps (20 episodes, deterministic policy), with the mean cumulative reward serving as the fitness value; after 20 generations of iteration, the optimal configuration is output. Phase 2—model training: PPO is re-initialized with the optimal hyperparameters and undergoes full policy learning in the training environment until convergence. Phase 3—generalization evaluation: the trained model is validated across multiple test environments encompassing different trajectory types, disturbance conditions, and scales.

The computational overhead of CCO–PPO is concentrated primarily in the optimization phase, with a total cost of $N\times \mathrm{MaxIt}\times 200,000=2.4\times 10^{7}$ training steps. All competing metaheuristic algorithms share this identical evaluation budget, ensuring that performance differences reflect search strategy efficiency rather than computational disparity; the results are summarized in Table 1. Additionally, BO incurs extra overhead for surrogate model maintenance (e.g., TPE) beyond the PPO training runs.

## 4. Experiments and Results

### 4.1. Experimental Environment Configuration

The experiments are implemented in Python 3.8 and PyTorch 1.12.0, with the simulation environment built on Gymnasium and executed on an Intel Xeon Gold 6248R CPU (3.0 GHz, 48 cores, Intel Corporation, Santa Clara, CA, USA) and NVIDIA A100 GPU (40 GB, NVIDIA Corporation, Santa Clara, CA, USA).

**UAV Dynamic Model.** A quadrotor UAV model is adopted with mass of 1.0kg, arm length of 0.225m, and inertia $I=\left[0.01,0.01,0.02\right]\;\mathrm{kg}\cdot m^{2}$, governed by the Newton–Euler equations, with single-motor thrust up to 15.0N, total thrust [24.0,60.0]N, and control frequency 50Hz (Δt=0.02s).

**Reference Trajectories and Disturbance Conditions.** Five reference trajectories and two disturbance conditions are designed to evaluate tracking performance and robustness, as detailed in Table 2.

The wind disturbance amplitude of 0.5 m/s^2^ corresponds to the median of Beaufort force 3 equivalent accelerations (0.3–0.8 m/s^2^), representing typical conditions in urban and agricultural UAV scenarios.

**Episode Settings.** Each episode has a maximum of 1000 steps (20 s), with initial position offset ±0.2 m, velocity 90–110% of target plus ±0.1 m/s noise, attitude ±0.03 rad, and angular velocity ±0.05 rad/s. An episode is terminated early if any of the following conditions is met: (1) the distance from the target point in the XY plane exceeds 80 m; (2) the flight altitude falls below 0.3m or exceeds 50m; (3) |ϕ|>0.95πrad or |θ|>0.95πrad.

#### 4.1.1. Reward Function Design

The reward function $r_{t}$ designed in this paper is a piecewise composite function consisting of eight sub-terms:

(1) $$r_{t}=r_{\mathrm{position}}+r_{\mathrm{height}}+r_{z\_\mathrm{bonus}}+r_{\mathrm{velocity}}+r_{\mathrm{vel}\_\mathrm{match}}+r_{\mathrm{attitude}}+r_{\mathrm{angular}}+r_{\mathrm{survive}}$$

##### Position Reward $r_{\mathrm{position}}$

A piecewise design uses exponential reward for $e_{p}<1.5\;m$ and linear penalty otherwise to prevent gradient vanishing:

$$r_{\mathrm{position}}=\left\{\begin{array}{cc} 50.0\times \exp(-2.0\cdot e_{p}), & e_{p}<1.5\;m\\ 50.0\times \exp\left(-3.0\right)-25.0\times \left(e_{p}-1.5\right), & e_{p}\geq 1.5\;m \end{array}\right.$$

##### Height Reward $r_{\mathrm{height}}$

A fixed reward of 20.0 is granted for z∈[3,25]m, with a quadratic penalty outside this range.

##### Height Tracking Reward $r_{z\_\mathrm{bonus}}$

Building upon the altitude safety constraint, this term further encourages precise vertical position control:

$$r_{z\_\mathrm{bonus}}=10.0\times \exp\left(-∥z-z_{\mathrm{target}}∥\right)$$

##### Velocity Penalty $r_{\mathrm{velocity}}$

A linear penalty on velocity deviation suppresses aggressive maneuvers:

$$r_{\mathrm{velocity}}=-3.0\times ∥v_{t}-v_{t}^{\mathrm{target}}∥$$

##### Velocity Matching Reward $r_{\mathrm{vel}\_\mathrm{match}}$

Acting in concert with the velocity penalty to form a dual constraint, this term further improves velocity tracking accuracy:

$$r_{\mathrm{vel}\_\mathrm{match}}=10.0\times \exp\left(-1.5\times ∥v_{t}-v_{t}^{\mathrm{target}}∥\right)$$

##### Attitude Penalty $r_{\mathrm{attitude}}$

Suppresses deviations of the roll and pitch angles to maintain flight stability:

$$r_{\mathrm{attitude}}=-1.0\times \left(\phi^{2}+\theta^{2}\right)$$

##### Angular Velocity Penalty $r_{\mathrm{angular}}$

Penalizes excessive angular velocity to promote smooth flight:

$$r_{\mathrm{angular}}=-0.2\times ∥\omega_{t}∥$$

##### Survival Reward $r_{\mathrm{survive}}$

A fixed value of 2.0, encouraging the UAV to sustain flight rather than terminate the episode prematurely.

The weighting coefficients were determined through systematic manual tuning guided by physical reasoning. Position reward (up to 50.0) carries the largest weight as the primary control objective; height reward (20.0) and height tracking bonus (10.0) jointly ensure altitude safety and vertical precision. The velocity penalty and matching reward (3.0 and 10.0) form a dual constraint suppressing aggressive maneuvers while improving velocity tracking. Attitude and angular velocity penalties (1.0 and 0.2) serve as soft stability constraints without dominating the position objective, and the survival reward (2.0) encourages episode completion. All weights are held fixed across all seven environments, and CCO–PPO achieves consistent sub-meter accuracy throughout, supporting the generalizability of this reward design.

In the above, the position error is $e_{p}=∥\Delta p_{t}∥$, ϕ and θ denote the roll and pitch angles, respectively, and $\omega_{t}$ is the body angular velocity vector. The total reward is clipped to the interval [−500,100].

#### 4.1.2. Action Space Mapping

The normalized action output by the policy network is $a_{t}\in {\left[-1,\;1\right]}^{4}$. The thrust command is mapped to $F=24.0+\frac{\left(\mathrm{thrust}\_\mathrm{cmd}+1\right)}{2}\times 36.0\;N$, and attitude and yaw rate commands are passed to the low-level controller with gain $k_{f}=0.5$.

### 4.2. Hyperparameter Optimization Algorithm Comparison Experiment

#### 4.2.1. Experimental Design

This experiment compares the CCO against the KEO, DBO, PSO, and BO in PPO hyperparameter optimization using a two-stage design: training environment optimization followed by multi-environment validation.

**Training Phase.** All five algorithms perform hyperparameter search in the circle_slow environment, which enables rapid and stable fitness evaluation. BO is implemented via the Optuna with the TPE sampler, sharing the same four-dimensional search space and evaluation budget of 120 trials as the other algorithms. Each algorithm is run with 5 different random seeds (42, 123, 456, 789, 999), outputting 5 optimal configurations in total.

**Validation Phase.** Configurations are validated in circle_slow and figure8-WN, with each tested 5 times per environment, yielding 25 tests per algorithm.

**Evaluation Metrics.** Performance is measured by mean cumulative reward and standard deviation, with Wilcoxon rank-sum tests (α=0.05) against CCO.

#### 4.2.2. Experimental Results

**Figure 3:**
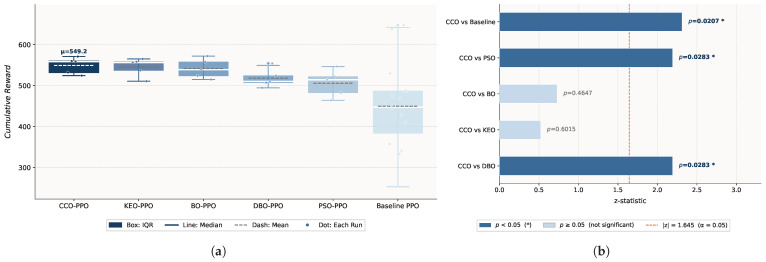
The experimental results for circle movements in slow mode: (**a**) boxplot of the cumulative reward distribution; (**b**) significance analysis of the performance metrics.

**The circle_slow Environment.** In circle_slow, CCO–PPO achieves the highest mean reward (549.20) and among the lowest standard deviations (17.75), with baseline PPO showing the largest variability (61.37), as shown in Figure 3. The CCO differs significantly from the DBO and PSO (p=0.0283,p=0.0305) but not from the KEO (p=0.6015) or baseline (p=0.5705).

BO–PPO achieves a mean reward of 541.58 (std = 21.05), with no significant difference from CCO–PPO (*p* = 0.4647) but significantly outperforming baseline PPO (*p* = 0.0207).

**Figure 4:**
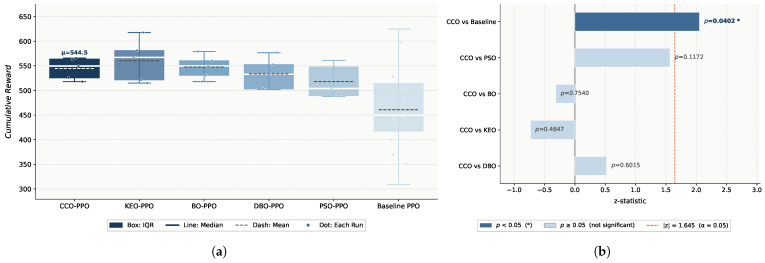
The experimental results for figure-eight movements under wind interference: (**a**) boxplot of the cumulative reward distribution; (**b**) significance analysis of the performance metrics.

**The figure8-WN Environment.** KEO–PPO achieves the highest mean reward (560.46) but with a standard deviation of 38.31; CCO–PPO ranks second (544.49, std=19.36), the lowest among optimized algorithms; DBO–PPO (533.49) and PSO–PPO (518.00) rank third and fourth, respectively; baseline PPO attains only 479.86 with a standard deviation of 53.32, significantly lower than all optimized algorithms. Significance testing shows that the CCO differs significantly from baseline (p=0.0078) but not from the DBO (p=0.6015), the KEO (p=0.4647), or PSO (p=0.1172), as shown in Figure 4.

In this environment, BO–PPO ranks second with a mean reward of 547.65 (std = 21.58), marginally above CCO–PPO (544.49) but without statistical significance (*p* = 0.7540).

#### 4.2.3. Result Analysis

Across both environments, the CCO achieves the best overall performance and stability: the highest mean reward in the simple environment (549.20), the lowest standard deviation in the complex environment (19.36), and the only algorithm with a statistically significant difference from the baseline under complex disturbance (p=0.0078). Although the KEO achieves the highest mean in the complex environment (560.46), its standard deviation (38.31) is nearly twice that of the CCO (19.36), indicating poor consistency across random seeds. PSO ranks last in both mean performance and stability, exhibiting limited search efficiency in the high-dimensional hyperparameter space. The DBO demonstrates conservative behavior with adequate local search but insufficient global exploration.

Environmental complexity influences performance rankings, yet the CCO consistently maintains stable performance. In the simple environment, the CCO shows no significant difference from the baseline. In the complex disturbance environment, CCO–PPO improves upon the baseline by approximately 13.5% (p=0.0078), demonstrating that fine-grained hyperparameter optimization substantially enhances policy robustness under non-stationary high-disturbance conditions. The notably large standard deviations of the baseline in both environments (61.37 and 53.32) further indicate the limited generalization capability of default parameters.

BO achieves performance comparable to the CCO in both environments (*p* = 0.4647, *p* = 0.7540); however, the CCO yields lower standard deviations in both environments (17.75 and 19.36 vs. 21.05 and 21.58), indicating superior consistency across random seeds. Moreover, BO’s reliance on sequential surrogate model construction limits parallelization, whereas the CCO’s population-based mechanism natively supports parallel evaluation, offering better scalability.

### 4.3. Ablation Study

#### 4.3.1. Experimental Design

To quantify the independent contribution of each hyperparameter, six ablation configurations are designed: baseline PPO (default values and reference), the CCO-ϵ (optimizing ϵ only), the CCO-lr (optimizing lr only), the CCO-lr,γ (optimizing lr and γ), the CCO-lr,ϵ (optimizing lr and ϵ), and the CCO (Full) (joint optimization of all four hyperparameters). Each configuration is independently run 5 times in the circle_slow and figure8-WN environments, with the CCO (Full) as the reference for Wilcoxon rank-sum tests (α=0.05). Baseline PPO results are taken directly from Section 4.2 (circle_slow: 527.65±61.37; figure8-WN: 479.86±53.32) to ensure consistency across experiments.

#### 4.3.2. Experimental Results

**Figure 5:**
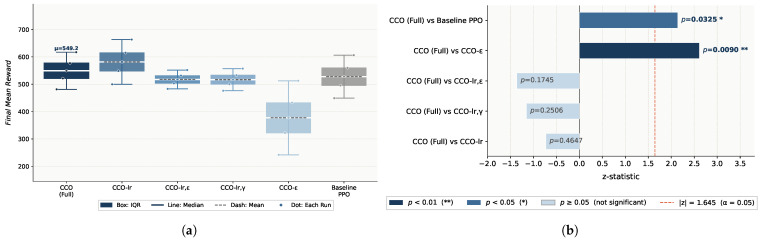
Ablation study results in the circle_slow (no disturbance) environment: (**a**) performance distribution of each hyperparameter optimization subset; (**b**) Wilcoxon rank-sum significance test against CCO (Full).

**The circle_slow environment.** The CCO (Full) achieves a mean reward of 549.20, significantly outperforming baseline PPO (527.65, *p* = 0.0325) and the CCO-ϵ (377.26, *p* = 0.0090). The CCO-lr yields the highest mean (581.51), with no significant difference from the CCO (Full) (*p* = 0.4647). Notably, the CCO-ϵ (377.26) falls below baseline PPO, as shown in Figure 5.

**Figure 6:**
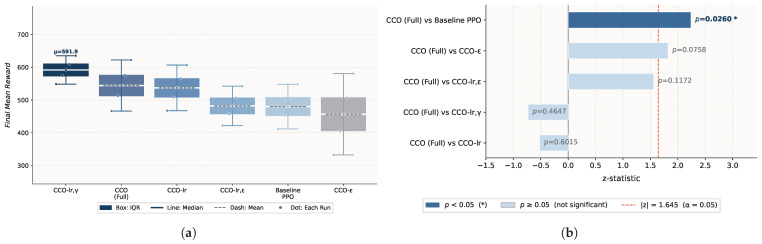
Ablation study results in the figure8-WN (wind disturbance + sensor noise) environment: (**a**) performance distribution of each hyperparameter optimization subset; (**b**) Wilcoxon rank-sum significance test against CCO (Full).

**The figure8-WN environment.** CCO (Full) achieves a mean reward of 544.49, significantly outperforming baseline PPO (479.86, *p* = 0.0260). The CCO-lr,γ yields the highest mean (591.93), marginally above the CCO (Full) but without statistical significance (*p* = 0.4647). The advantage of the CCO-lr narrows considerably (537.08), the CCO-lr,ϵ (482.07) underperforms relative to the CCO-lr,γ, and the CCO-ϵ (456.56) remains comparable to baseline PPO (*p* = 0.0758), as shown in Figure 6.

#### 4.3.3. Result Analysis

The learning rate lr is the most influential single hyperparameter; optimizing lr alone improves the mean reward by approximately 10% in the simple environment. In circle_slow, CCO-lr (581.51) slightly exceeds CCO (Full) (549.20), attributable to the higher sampling density of one-dimensional search under the same evaluation budget. In figure8-WN, however, the trend reverses: the CCO (Full) surpasses all partial optimization variants, demonstrating that interparameter coupling effects become pronounced under high-disturbance conditions and that joint optimization of all hyperparameters is essential.

The discount factor γ plays a critical supplementary role in disturbance environments: the CCO-lr,γ achieves the highest mean (591.93), indicating that precise adjustment of long-horizon return estimation becomes particularly important under high-noise conditions. The CCO-lr,ϵ underperforms relative to the CCO-lr,γ in the disturbance environment, confirming that uncoordinated optimization of lr and ϵ produces counteracting effects. The CCO-ϵ falls significantly below baseline PPO in the simple environment (*p* = 0.0090), demonstrating that effective ϵ search requires coordinated lr configuration.

Overall, the CCO (Full) significantly outperforms baseline PPO in both environments while maintaining the most stable mean-variance balance across all variants, validating the robustness of full-parameter joint optimization.

### 4.4. Comprehensive Performance Validation Experiment

#### 4.4.1. Experimental Design

This experiment validates the generalization capability and robustness of CCO–PPO across 7 test environments of progressively increasing difficulty.

**Test Environment Design.** The 7 environments are divided into three levels of difficulty. The first level comprises baseline environments (circle_slow, helix_up and figure8), with no disturbances, evaluating basic tracking across different trajectory geometries. The second level comprises disturbance environments (figure8-W and figure8-WN), evaluating robustness under wind and sensor noise. The third level comprises large-scale environments (circle_slow_large and figure8_large), with trajectory scale expanded from 5 m to 20 m, testing policy transfer to unseen scenarios.

**Comparison Algorithm Configuration.** CCO–PPO employs the 5 optimal configurations from the hyperparameter optimization experiment (Section 4.2), each tested 5 times per environment (n=5). Training steps are uniformly set to 200,000, with consistent network architectures (SAC and TD3 do not use LSTM).

As a background reference, a cascaded PID controller yields an average episode length of only 65–75 steps and a mean tracking error of approximately 18–22 m in circle_slow and figure8, consistent with the limited robustness of traditional controllers under nonlinear dynamics [1,2]. As PID involves no training process, it is excluded from Table 3 and serves only as an order-of-magnitude reference.

**Evaluation Metrics.** Mean tracking error (in meters) serves as the primary performance metric, which is more intuitive than cumulative reward and facilitates cross-environment comparison. Using CCO–PPO as the reference, the Wilcoxon rank-sum test (α=0.05) is applied separately against each of the other three algorithms to assess statistical significance.

#### 4.4.2. Experimental Results

Table 3 summarizes the tracking errors and significance test results for the four algorithms across the seven test environments. CCO–PPO achieves the lowest tracking error in all environments.

**Baseline Trajectory Environments.** CCO–PPO achieves tracking errors of 0.7222 m, 0.7550 m, and 0.7132 m in the three environments, respectively—all being the lowest. Compared to baseline PPO, the improvement margin increases with trajectory complexity: 4.6% in circle_slow (p=0.0264), 10.7% in helix_up (p=0.0011), and 13.4% in figure8 (p<0.0001). CCO–PPO significantly outperforms SAC in all three environments (p<0.0113), while TD3 shows no significant difference from CCO–PPO.

**Disturbance Environments.** CCO–PPO achieves tracking errors of 0.7149 m and 0.7232 m in figure8-W and figure8-WN, respectively, representing negligible degradation compared to the disturbance-free figure8 (0.7132 m), with increases of only 0.2% and 1.4%; by contrast, baseline PPO deteriorates from 0.8234 m to 0.8912 m (an increase of 8.2%). In figure8-W, CCO–PPO improves upon baseline by 16.5% (p=0.0001) and achieves a significant advantage over TD3 for the first time (p=0.0369); in figure8-WN, the differences between CCO–PPO and all three comparison algorithms are statistically significant (p<0.0008), with the improvement over baseline reaching 18.8%—the largest across all environments.

**Generalization Environments.** In circle_slow_large and figure8_large, CCO–PPO achieves tracking errors of 0.7399 m and 0.7433 m, respectively—representing increases of only 2.5% and 4.2% compared to the corresponding small-scale environments (0.7222 m and 0.7132 m), with minimal generalization loss. In circle_slow_large, CCO–PPO shows no significant difference from baseline (p=0.3351) but significantly outperforms SAC (p=0.0082) and TD3 (p=0.0155); in figure8_large, CCO–PPO differs significantly from all comparison algorithms (p≤0.0008), improving upon baseline by 8.5%.

Across all 21 significance tests, CCO–PPO achieves significance against baseline in 6/7 cases, against SAC in 7/7 cases, and against TD3 in 5/7 cases, yielding an overall significance rate of 85.7%.

#### 4.4.3. Result Analysis

**Baseline Environments.** Under disturbance-free conditions, the performance gaps among the algorithms are relatively small, and the advantage of CCO–PPO grows with increasing trajectory complexity (4.6% improvement in circle_slow; 13.4% in figure8), indicating that hyperparameter optimization yields greater value in dynamically complex scenarios. TD3 shows no significant difference from CCO–PPO in baseline environments, reflecting the suitability of deterministic policies for continuous control tasks.

**Disturbance Environments.** Baseline PPO exhibits an 8.2% degradation in tracking error upon the introduction of disturbances, while CCO–PPO remains nearly unaffected (increase ≤1.4%), demonstrating that optimized hyperparameters substantially enhance policy robustness. The improvement margins of CCO–PPO in disturbance environments (16.5–18.8%) are markedly higher than in baseline environments, and CCO–PPO achieves a significant advantage over TD3 for the first time, indicating that on-policy methods with targeted hyperparameter optimization exhibit superior adaptability under non-stationary conditions compared to off-policy methods with default configurations.

**Generalization Environments.** After transferring from the 5 m training scale to the 20 m evaluation scale, CCO–PPO incurs a tracking error increase of only 2.5–4.2%, demonstrating good scale invariance of the CCO optimization results.

**Overall Evaluation.** CCO–PPO achieves a significance rate of 85.7% across 21 significance tests, with performance advantages increasing monotonically with environmental complexity, validating the effectiveness and practical value of metaheuristic hyperparameter optimization for complex high-disturbance UAV trajectory tracking tasks.

From a practical standpoint, CCO–PPO maintains a stable mean tracking error of 0.72–0.74 m across all the test environments, meeting the sub-meter accuracy requirements of applications such as precision agriculture and indoor navigation. Under disturbance conditions, the error reduction of approximately 15–19 cm relative to baseline PPO can meaningfully improve mission success rates and safety margins in obstacle-dense or spatially constrained scenarios.

The CCO hyperparameter optimization phase requires $2.4\times 10^{7}$ training steps as a one-time offline investment; once the optimal configuration is identified, the trained PPO policy executes at 50 Hz with inference latency well within the real-time requirements for quadrotor control. The offline nature of the optimization ensures that deployment on embedded flight controllers imposes no additional computational overhead at runtime.

## 5. Conclusions

This paper proposes the CCO–PPO framework, which integrates the cuckoo catfish optimizer with PPO to address the time-consuming and unstable nature of manual hyperparameter tuning in UAV trajectory tracking through automated hyperparameter search. Experiments across seven test environments demonstrate that CCO–PPO achieves the lowest tracking error in all the scenarios, with performance advantages that grow monotonically with task complexity—reaching an improvement of 18.8% over baseline PPO under the challenging combined conditions of wind disturbance and sensor noise while also exhibiting favorable scale generalization capability. Ablation studies further demonstrate that joint optimization of all four hyperparameters is essential under high-disturbance conditions, and comparisons with BO confirm the CCO’s superior stability and scalability as the core optimizer. These results indicate that systematic hyperparameter optimization can yield substantial performance gains in high-disturbance, high-complexity scenarios and that CCO–PPO provides a practical and scalable trajectory tracking solution for real-world UAV autonomous flight systems.

Future work will explore two directions: evaluating CCO–PPO under continuously varying disturbance intensities—including higher wind speeds and stronger sensor noise—to characterize its robustness boundary more completely and investigating generalization across heterogeneous UAV platforms with varying mass and inertia parameters to assess the transferability of the optimized hyperparameters.

## Figures and Tables

**Table 1:** Algorithm performance comparison (unit: steps or evaluations).

Algorithm	Evaluations	Total Steps	circle_slow Mean	figure8-WN Mean	figure8-WN Std
CCO–PPO	120	$2.4\times 10^{7}$	549.20	544.49	19.36
BO–PPO	120	$2.4\times 10^{7}$	541.58	547.65	21.58
KEO–PPO	120	$2.4\times 10^{7}$	545.52	560.46	38.31
DBO–PPO	120	$2.4\times 10^{7}$	518.09	533.49	28.81
PSO–PPO	120	$2.4\times 10^{7}$	505.88	518.00	30.55
Baseline PPO	—	—	527.65	479.86	53.32

Note: “—” indicates no data available. Units for total steps are $10^{7}$ steps; other metrics are unitless.

**Table 2:** Reference trajectory and disturbance condition parameters.

Trajectory ID	Radius/Size	Period	Center (x, y)	Altitude	Disturbance Conditions
circle_slow	5 m	20 s	(0, 0) m	10 m	—
helix_up	5 m	20 s	(0, 0) m	5→15 m, $v_{z}=$ 0.5 m/s	—
figure8	5 m	20 s	(0, 0) m	10 m	—
figure8-W	5 m	20 s	(0, 0) m	10 m	Random wind disturbance, 0.5 m/s^2^
figure8-WN	5 m	20 s	(0, 0) m	10 m	Wind disturbance + sensor noise (σ=0.01)
circle_slow_large	20 m	40 s	(0, 0) m	15 m	—
figure8_large	20 m	40 s	(0, 0) m	15 m	—

**Table 3:** Tracking error comparison and significance tests across seven environments (unit: meters).

Environment	CCO–PPO	Baseline PPO	SAC	TD3	CCO vs. Baseline	CCO vs. SAC	CCO vs. TD3
circle_slow	0.7222	0.7573	0.7891	0.7312	p=0.0264 *	p=0.0133 *	p=0.6764
helix_up	0.7550	0.8456	0.8723	0.7889	p=0.0011 **	p=0.0008 **	p=0.1403
figure8	0.7132	0.8234	0.7645	0.7423	p<0.0001 **	p=0.0113 *	p=0.1127
figure8_wind	0.7149	0.8567	0.7889	0.7656	p=0.0001 **	p=0.0029 **	p=0.0369 *
figure8_noise	0.7232	0.8912	0.8123	0.7923	p=0.0008 **	p=0.0005 **	p=0.0006 **
circle_slow_large	0.7399	0.7634	0.8234	0.7989	p=0.3351	p=0.0082 **	p=0.0155 *
figure8_large	0.7433	0.8123	0.7912	0.7845	p=0.0050 **	p=0.0322 *	p=0.0242 *

Note: The last three columns report *p*-values from Wilcoxon rank-sum tests. * *p* < 0.05; ** *p* < 0.01.

## Data Availability

The raw data supporting the conclusions of this article will be made available by the authors on request.

## References

[1] Bouabdallah S., Noth A., Siegwart R. PID vs LQ control techniques applied to an indoor micro quadrotor. Proceedings of the IEEE/RSJ International Conference on Intelligent Robots and Systems.

[2] Nascimento T.P., Saska M. (2019). Position and attitude control of multi-rotor aerial vehicles: A survey. Annu. Rev. Control.

[3] Kaufmann E., Bauersfeld L., Loquercio A., Müller M., Koltun V., Scaramuzza D. (2023). Champion-level drone racing using deep reinforcement learning. Nature.

[4] Schulman J., Wolski F., Dhariwal P., Radford A., Klimov O. (2017). Proximal policy optimization algorithms. arXiv.

[5] Haarnoja T., Zhou A., Abbeel P., Levine S. Soft actor-critic: Off-policy maximum entropy deep reinforcement learning with a stochastic actor. Proceedings of the International Conference on Machine Learning.

[6] Fujimoto S., van Hoof H., Meger D. Addressing function approximation error in actor-critic methods. Proceedings of the International Conference on Machine Learning.

[7] Andrychowicz M., Raichuk A., Stańczyk P., Orsini M., Girgin S., Marinier R., Hussenot L., Geist M., Pietquin O., Michalski M. (2020). What matters in on-policy reinforcement learning? A large-scale empirical study. arXiv.

[8] Hu G., Huang F., Shu B., Zhang Z., Li Y. (2025). MAHACO: Multi-algorithm hybrid ant colony optimizer for 3D path planning of a group of UAVs. Inf. Sci..

[9] Santos T., Silva L., Cossetin Neto A., Pignaton de Freitas E. (2025). Solving pathfinding problems in cubic grids using 3D neighborhood extension. Expert Syst. Appl..

[10] Xu T., Chen C. (2025). DBO-AWOA: An adaptive whale optimization algorithm for global optimization and UAV 3D path planning. Sensors.

[11] Bergstra J., Bengio Y. (2012). Random search for hyper-parameter optimization. J. Mach. Learn. Res..

[12] Shahriari B., Swersky K., Wang Z., Adams R.P., de Freitas N. (2016). Taking the human out of the loop: A review of Bayesian optimization. Proc. IEEE.

[13] Jaderberg M., Dalibard V., Osindero S., Czarnecki W.M., Donahue J., Razavi A., Vinyals O., Green T., Dunning I., Simonyan K. (2017). Population based training of neural networks. arXiv.

[14] Kennedy J., Eberhart R. Particle swarm optimization. Proceedings of the IEEE International Conference on Neural Networks.

[15] Storn R., Price K. (1997). Differential evolution—A simple and efficient heuristic for global optimization over continuous spaces. J. Glob. Optim..

[16] Xue J., Shen B. (2023). Dung beetle optimizer: A new meta-heuristic algorithm for global optimization. J. Supercomput..

[17] Koch W., Mancuso R., West R., Bestavros A. (2019). Reinforcement learning for UAV attitude control. ACM Trans. Cyber-Phys. Syst..

[18] Molchanov A., Chen T., Hönig W., Preiss J.A., Ayanian N., Sukhatme G.S. (2019). Sim-to-(multi)-real: Transfer of low-level robust control policies to multiple quadrotors. arXiv.

[19] Schulman J., Moritz P., Levine S., Jordan M., Abbeel P. (2015). High-dimensional continuous control using generalized advantage estimation. arXiv.

[20] Wang T.-L., Gu S.-W., Liu R.-J., Chen L.-Q., Wang Z., Zeng Z.-Q. (2025). Cuckoo catfish optimizer: A new meta-heuristic optimization algorithm. Artif. Intell. Rev..

